# Histone deacetylase 3 promotes hypoxia-induced human pulmonary arterial smooth muscle cell proliferation by modulating the CSF2-JAK2-STAT5 signaling pathway

**DOI:** 10.1007/s13577-026-01348-6

**Published:** 2026-01-16

**Authors:** Jie Zhang, Youfei Fan, Yanting Gao, Youpeng Jin

**Affiliations:** 1 Department of Pediatrics, Shandong Provincial Hospital Affiliated to Shandong First Medical University, Jinan, 250014 People’s Republic of China; 2 Department of Pediatric Intensive Care Unit, The Second Affiliated Hospital of Shandong First Medical University, Taian, 271000 People’s Republic of China

**Keywords:** Histone deacetylase 3, Pulmonary arterial hypertension, Hypoxia, Human pulmonary arterial smooth muscle cells, CSF2, Jak-Stat signaling

## Abstract

The growth of human pulmonary arterial smooth muscle cells (hPASMCs) is one of the key contributors to vascular remodeling in pulmonary arterial hypertension (PAH). Although histone deacetylase 3 (HDAC3) has been implicated in acute lung injury and pulmonary fibrosis, its role in hypoxia-induced PAH remains unclear. Here, the function and associated mechanisms of HDAC3 in hypoxia-induced hPASMC proliferation were investigated. A hypoxia-induced hPASMC model was constructed to evaluate the role of HDAC3 in cell proliferation under hypoxic conditions. The effects of HDAC3 siRNA and ruxolitinib, a JAK pathway inhibitor, were assessed to explore the regulatory mechanism of HDAC3 in vascular remodeling. Hypoxia significantly upregulated both HDAC3 mRNA and protein. Inhibition of HDAC3 attenuated hypoxia-induced proliferation in hPASMCs. Moreover, HDAC3 inhibition downregulated CSF2 and suppressed proliferation by inactivating the JAK2/STAT5 axis. In contrast, HDAC3 overexpression enhanced CSF2 expression, activated JAK2/STAT5, and promoted hPASMCs’ proliferation under hypoxia. Notably, the pro-proliferative and pathway-activating effects of HDAC3 overexpression were reversed by CSF2 silencing or ruxolitinib treatment. HDAC3 plays a key role in hypoxia-induced hPASMC dysfunction. Its inhibition mitigates aberrant proliferation through a CSF2-dependent inactivation of the JAK2/STAT5 pathway under hypoxia. These results indicate the potential of using HDAC3 for treating hypoxia-induced PAH.

**Supplementary Information:**

The online version contains supplementary material available at 10.1007/s13577-026-01348-6.

## Introduction

Pulmonary arterial hypertension (PAH) is characterized by a mean pulmonary arterial pressure > 20 mmHg at rest. Pulmonary vascular remodeling, a hallmark of PAH, involves progressive increases in pulmonary arterial pressure accompanied by vascular resistance, and can lead to right heart failure and subsequent death [1]. Changes in the proliferation, apoptosis, and chronic inflammatory responses of human pulmonary arterial smooth muscle cells (hPASMCs) are linked to vascular remodeling and luminal obstruction [2–4]. Notably, a recent study by Jin et al. demonstrated that the Sonic hedgehog (SHH) signaling inhibitor cyclopamine ameliorates PAH by regulating the BMPR2 pathway, highlighting the therapeutic potential of targeting developmental signaling cascades in pulmonary vascular remodeling [5]. Although hypoxia-inducible factor (HIF)−2α has demonstrated therapeutic potential in ameliorating PAH and right ventricular remodeling in rodent models under both preventive and reversal treatment protocols [3], the precise mechanisms underlying hPASMC dysfunction under hypoxic conditions remain poorly understood.

There are currently 18 known histone deacetylases (HDACs) in mammals, identified from yeast homologs. Histone deacetylase 3 (HDAC3), a class I HDAC, exists in four splicing variants: HD3α, HD3β, HD3γ, and HD3δ [6]. HDAC3 not only regulates gene transcription via histone deacetylation but also modifies non-histone proteins, including cytoplasmic and mitochondrial proteins, through post-translational mechanisms. Previous studies have reported increased HDAC1 and HDAC5 expression in remodeled vessels of patients with idiopathic PAH (IPAH) and demonstrated that HDAC inhibitors could reverse hypoxia-induced PAH in rat models and in hPASMCs derived from IPAH patients by exerting antiproliferative and anti-inflammatory effects [7–9]. However, the specific role of HDAC3 in this context remains unclear. Under hypoxic conditions, HDAC3 has been shown to exacerbate pulmonary fibrosis by promoting epithelial–mesenchymal transition in alveolar epithelial cells [10], but whether HDAC3 inhibition can attenuate hPASMC proliferation and cytokine dysregulation in hypoxia has not been established.

PAH is characterized by aberrant tumor-like signaling pathways that drive excessive cellular proliferation, survival, inflammation, and extracellular matrix dysregulation [3, 11, 12]. The strongly conserved JAK/STAT axis mediates the effects of cytokines, growth factors, and interferons [4, 13]. In pulmonary fibrosis and PAH, recent studies found co-localization of α-smooth muscle actin (α-SMA) with JAK2/p-STAT3 in endothelial cells and suggested that the JAK/STAT pathway played a key role in vascular remolding [14]. Pharmacological inhibition with ruxolitinib, a JAK inhibitor, has been shown to suppress cell proliferation and partially reverse PAH in preclinical models [4, 15, 16]. Furthermore, this pathway mediates downstream signaling of numerous proinflammatory cytokines, including interleukin-6, fibroblast growth factor, and four-and-a-half LIM domains protein 1 (FHL1), among others [17–20]. Increased or adventitial perivascular accumulation of monocytes/macrophages and elevated expression of CSF2 have been consistently observed in both PAH patients and hypoxia-induced PAH models [21]. Furthermore, PASMCs have been shown to directly secrete multiple proinflammatory mediators that exacerbate pulmonary artery remodeling [22] and chronic inflammation, contributing to pulmonary artery remodeling and PAH progression [23, 24]. In macrophages, HDAC3 knockout led to reduced expression of STAT1 and STAT2, resulting in defective antiviral responses in both cellular and animal models [25]. Moreover, ruxolitinib has been reported to suppress osteoclast activity in Hdac3 conditional knockout mice [26]. However, the potential interaction between HDAC3 and the JAK/STAT signaling axis in hypoxia-induced hPASMC dysfunction remains unknown.

Here, the function of HDAC3 in hypoxia-induced hPASMC dysfunction was investigated, together with a determination of whether HDAC3 contributes to vascular remodeling via the CSF2-mediated JAK2/STAT5 axis. The results indicate the potential of targeting HDAC3 for treating PAH resulting from hypoxia.

## Materials and methods

### Cell culture and treatment

The cell lines hPASMCs present in this study were obtained from iCell Bioscience Inc. (HUM-iCell-a009, China) and were grown in an iCell Primary Smooth Muscle Cell Low Serum Culture System (PriMed-iCell-004, China) at 37 °C with 5% CO_2_. Cells were grown until 70–80% confluent. Before treatment, cells were synchronized by incubation in a culture media with 1% FBS for 24 h. The hypoxia group was incubated under hypoxic conditions (5% CO_2,_ 5% O_2_, and 90% N_2_) for 48 h, while the normoxia control group was grown with 5% CO_2,_ 21% O_2_, and 74% N_2_, following established protocols [27]. At the end of treatment, hPASMCs were harvested for immunofluorescence staining and molecular analyses. Only cells at passages less than 10 were used for all experiments.

### siRNA transfections in hPASMCs

Small interfering RNAs (siRNAs) targeting human HDAC3 and CSF2, along with the corresponding transfection reagents, were procured from Tsingke Biotech Co., Ltd. (Beijing, China). hPASMCs were transfected with HDAC3-specific siRNA (HDAC3-KD), CSF2-specific siRNA (CSF2-KD), or scrambled negative control siRNA (NC-KD), as directed. The knockdown efficiency of three different siRNA sequences for HDAC3 and CSF2 was evaluated by qRT-PCR. The siRNA sequence demonstrating the highest knockdown efficiency selected for subsequent experiments is as follows: Sense, 5ʹ- GUUAUACUGUCCGAAAUGU-3ʹ; Antisense, 5ʹ-ACAUUUCGGACAGUAUAAC-3ʹ. The sequences of the siRNA used for CSF2 knockdown are as follows: Sense, 5ʹ- GCAACCCAGAUUAUCACCU-3ʹ; Antisense, 5ʹ-AGGUGAUAAUCUGGGUUGC-3ʹ. The scrambled siRNA was used as the negative control group.

### RNA-sequencing analysis

The purity of the RNA was assessed using NanoPhotometer® (IMPLEN, USA), while its concentration was determined using an Agilent 2100 Bioanalyzer using the RNA Nano 6000 Assay Kit (Agilent Technologies, USA). For each sample, 1–3 µg of total RNA was utilized as input material to prepare sequencing libraries while employing the VAHTS Universal V6 RNA-seq Library Prep Kit for Illumina® (NR604-01/02), as directed. Sequences were attributed to each sample by adding the index codes. Differential gene expression was analyzed using the DESeq286 package, comparing HDAC3-KD hPASMCs with the NC-KD control group. Genes with an absolute log_2_ fold change ≥ 1.5 and an adjusted *P* < 0.05 were deemed differentially expressed. Differentially expressed genes (DEGs) identified in both experimental replicates were retained for further analysis.

The functions of the DEGs were examined by KEGG enrichment using clusterProfiler in R, with *P* < 0.05 recognized as significant. Further gene set enrichment analysis (GSEA) was performed on significantly enriched genes which were ranked according to the criterion of (log_2_ fold change) × (− log_10_ FDR). Leading edge analysis was performed using GSEA results to identify specific genes and pathways contributing most strongly to the enrichment signals, allowing the identification of nodal genes within significantly upregulated or downregulated pathways.

### RNA isolation and qRT-PCR

Total RNA was extracted from hPASMCs using TRIzol (15596026CN, Invitrogen, USA) as directed. RNA was reverse transcribed into cDNA using a kit (AG11706, Accurate Biotechnology Co., Ltd., China). qRT-PCR was performed using the SYBR® Premix Ex Taq™ II kit (AG11701, Accurate Biotechnology Co., Ltd.) on a LightCycler 480 system (Roche, Switzerland). All reactions were performed in triplicate, and each experiment was repeated independently more than three times. Relative gene expression was determined against GAPDH using the 2^–ΔΔCt^ method. The primer sequences used for amplification are provided in Table 1.

**Table 1:** Primer sequences

Gene	Primer sequence (5’−3’)
HDAC3-F	ATGCTGAACCATGCACCTAGT
HDAC3-R	AAGTAGGCTGAAGTCCCTGCT
α-SMA-F	TGCAACATGGAAGGTATTGC
α-SMA-R	TTCACACAGCACCAAGC
CSF2-F	CATGATGGCCAGCCACTACAA
CSF2-R	ACTGGCTCCCAGCAGTCAAAG
CSF3-F	GCTTGAGCCAACTCCATAGCG
CSF3-R	CAGATGGTGGTGGCAAAGTCG
FHL1-F	GCCAAGAAGTGTGCTGGATG
FHL1-R	GGCCAGATTCACGGAGCATT
LIF-F	CCCAACAACCTGGACAAGCTA
LIF-R	AAGGTACACGACTATGCGGT
PRLR-F	GGACTTCCTACCAATTATTCACTG
PRLR-R	CACATGGAGGTGTACTGCTT
GAPDH-F	GAAATCCCATCACCATCTTCCAGG
GAPDH-R	GAGCCCCAGCCTTCTCCATG

### Western blotting

Separation of equal amounts of protein was carried from hPASMC lysates using 12% SDS-PAGE gels, followed by transferring them onto polyvinylidene fluoride membranes. Following blocking with 1% BSA, membranes were incubated with one of the following antibodies: monoclonal rabbit anti-HDAC3 antibody (1:1000; Ab32369, Abcam, Cambridge, United Kingdom), monoclonal rabbit anti-α-SMA antibody (1:2000; GB111364, Service bio, Wuhan, Hubei, China), polyclonal rabbit anti-GM-CSF/CSF2 antibody(1:1000; PA5-96,512, Thermo Fisher Scientific, Waltham, MA, USA), GB114197, Servicebio, monoclonal rabbit anti-PCNA (1:1000, A12427, AbClonal Technology, Wuhan, Hubei, China), polyclonal rabbit anti-JAK2 (1:500, A7694, AbClonal Technology, Wuhan, Hubei, China), polyclonal rabbit anti-Phospho-JAK2-Y1007/1008 (1:300, A0531, AbClonal Technology, Wuhan, Hubei, China), polyclonal rabbit anti-STAT5 (1:1000, A5029, AbClonal Technology, Wuhan, Hubei, China), polyclonal rabbit anti-Phospho-STAT5-Y694 (1:300, AP0887, AbClonal Technology, Wuhan, Hubei, China), or monoclonal rabbit anti-GAPDH (1:5000, 60,004–1-lg, Proteintech Group Inc, Chicago, IL, USA), followed by the matched secondary antibodies (Proteintech Group Inc., Chicago, IL, USA). The quantification of band intensity upon western blot analysis was conducted using NIH Image software (ProteinSimple, Santa Clara, CA, USA). Positive blots were visualized using ECL-Plus™ (Amersham, GE Healthcare, Waukesha, WI, USA).

### MTT assays

Following trypsin (C0201, Beyotime, Shanghai, China) digestion, hPASMCs were counted using a hemocytometer and diluted to a final concentration of 5 × 10^4^ cells/mL in complete medium. The cells (100 μL/well) were inoculated in 96-well plates and grown for 24 h before exposure to hypoxia (5% O_2_) and transfection, followed by incubation for another 48 h. Ten microliters of 5 mg/mL MTT were then introduced to individual wells, followed by incubation for 4 h away from light. Following removal of the supernatants, the formazan crystals were solubilized in DMSO and absorbances were read at 490 nm using a SpectraMax® iD3 Multi-Mode Microplate Reader (San Jose, California, USA).

### BrdU staining

hPASMCs were incubated with 50 μM BrdU (ST1056, Beyotime, Shanghai, China) for 1 h, then fixed with 4% paraformaldehyde and permeabilized with 0.1% Triton X-100. DNA denaturation was achieved using 2 M HCl, and neutralized with 0.1 M sodium borate (pH 8.5). After blocking with 5% normal goat serum, cells were incubated overnight at 4 °C with anti-BrdU antibody (1:1000, B2531, Sigma). The next day, cells were incubated for 1 h at 37 °C with rhodamine-conjugated secondary antibody (1:50, ZF0313, ZSGB-BIO, Beijing, China). Coverslips were washed, mounted with an antifade mounting medium, and visualized under a fluorescence microscope. BrdU-positive cells were counted in at least 100 cells from five randomly selected fields at 400 × magnification, with results shown as percentages of the total cells.

### Statistical analysis

Statistical analysis was performed with GraphPad Prism (version 9.0). The data are presented as the means ± standard deviations (SDs). Student’s *t*-test was used for comparisons between two groups. For comparisons among more than two groups: one-way ANOVA followed by LSD (Least Significant Difference) test was used for single-variable multiple-group comparisons. *P* < 0.05 was considered to indicate a significant difference.

## Results

### Hypoxia promoted HDAC3 upregulation and hPASMC proliferation

**Fig. 1:**
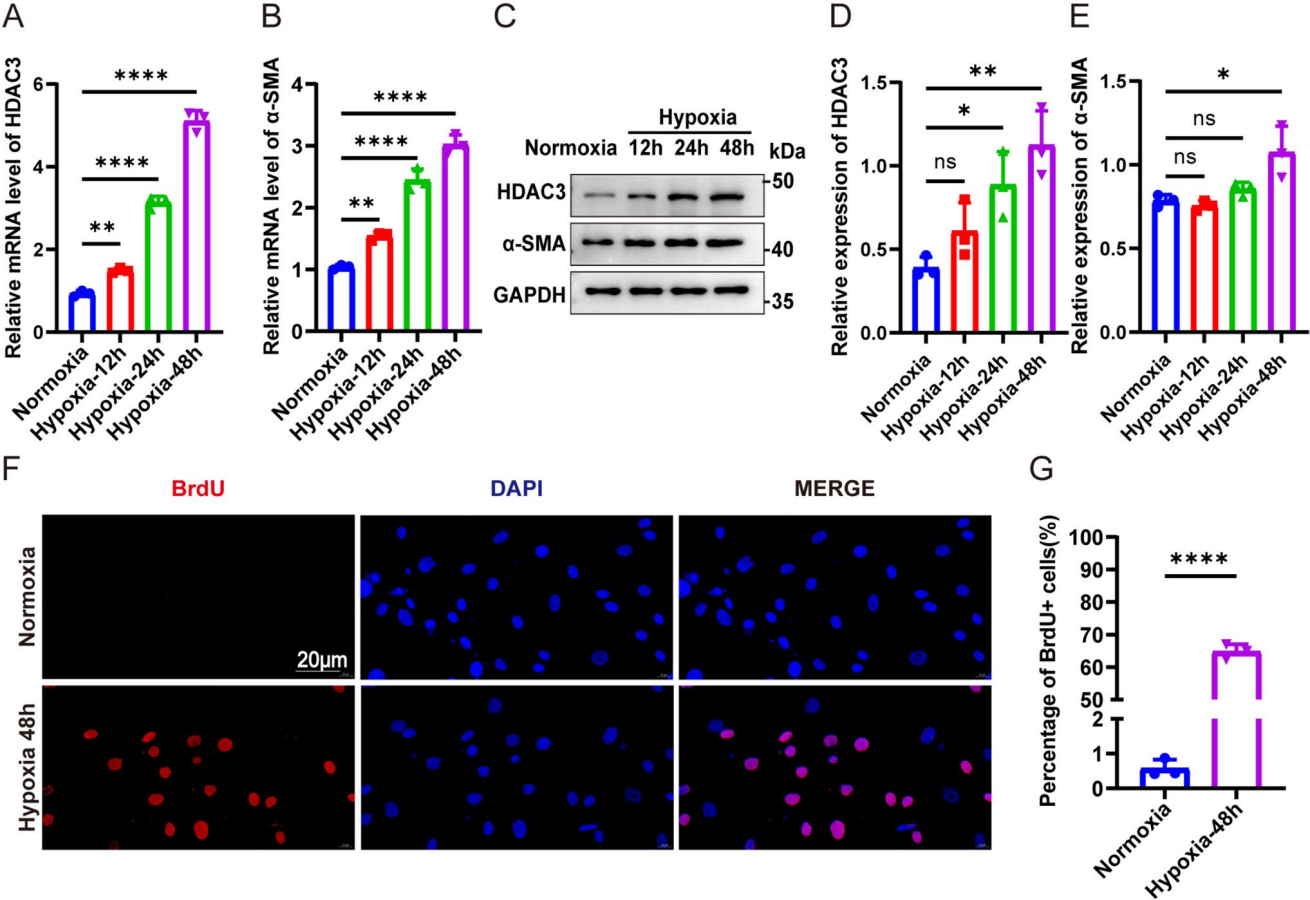
Hypoxia promoted HDAC3 upregulation and hPASMCs’ proliferation. The mRNA levels of HDAC3 (**A**) and α-SMA (**B**) under normoxia and hypoxia for 12 h, 24 h, and 48 h. **C** Western blot analysis of HDAC3 and α-SMA under normoxia and hypoxia for 12 h, 24 h, and 48 h. Representative Western blot and relative quantitative analyses of HDAC3 (**D**) and α-SMA (**E**) expression. **F** Immunofluorescent detection of BrdU-labeled cells (red) in hPASMCs under normoxia and hypoxia conditions for 48 h. The data are shown as the mean ± SD of at least three biologically independent replicates. Multiple-group comparisons were analyzed by one-way ANOVA followed by LSD post hoc test. *P < 0.05, **P < 0.01, ***P < 0.001

To investigate whether HDAC3 expression is affected by hypoxia and whether it contributes to hPASMC proliferation, cells were treated with normoxic or hypoxic conditions for 48 h (Fig. 1). Both qRT-PCR and western blot analyses showed increased HDAC3 mRNA (Fig. 1A) and protein (Fig. 1C, D) expression levels in a time-dependent manner under hypoxia, with the greatest upregulation seen at 48 h. Therefore, 48 h of hypoxic exposure was selected for subsequent experiments. To evaluate the proliferation of hPASMCs, α-SMA expression and BrdU incorporation were assessed. Hypoxia significantly increased both α-SMA mRNA and protein expression (Figs. 1B, E), as well as the proportion of BrdU-positive cells (Fig. 1F, G), indicating increased cell proliferation.

### HDAC3 promoted hypoxia-induced hPASMC proliferation

**Fig. 2:**
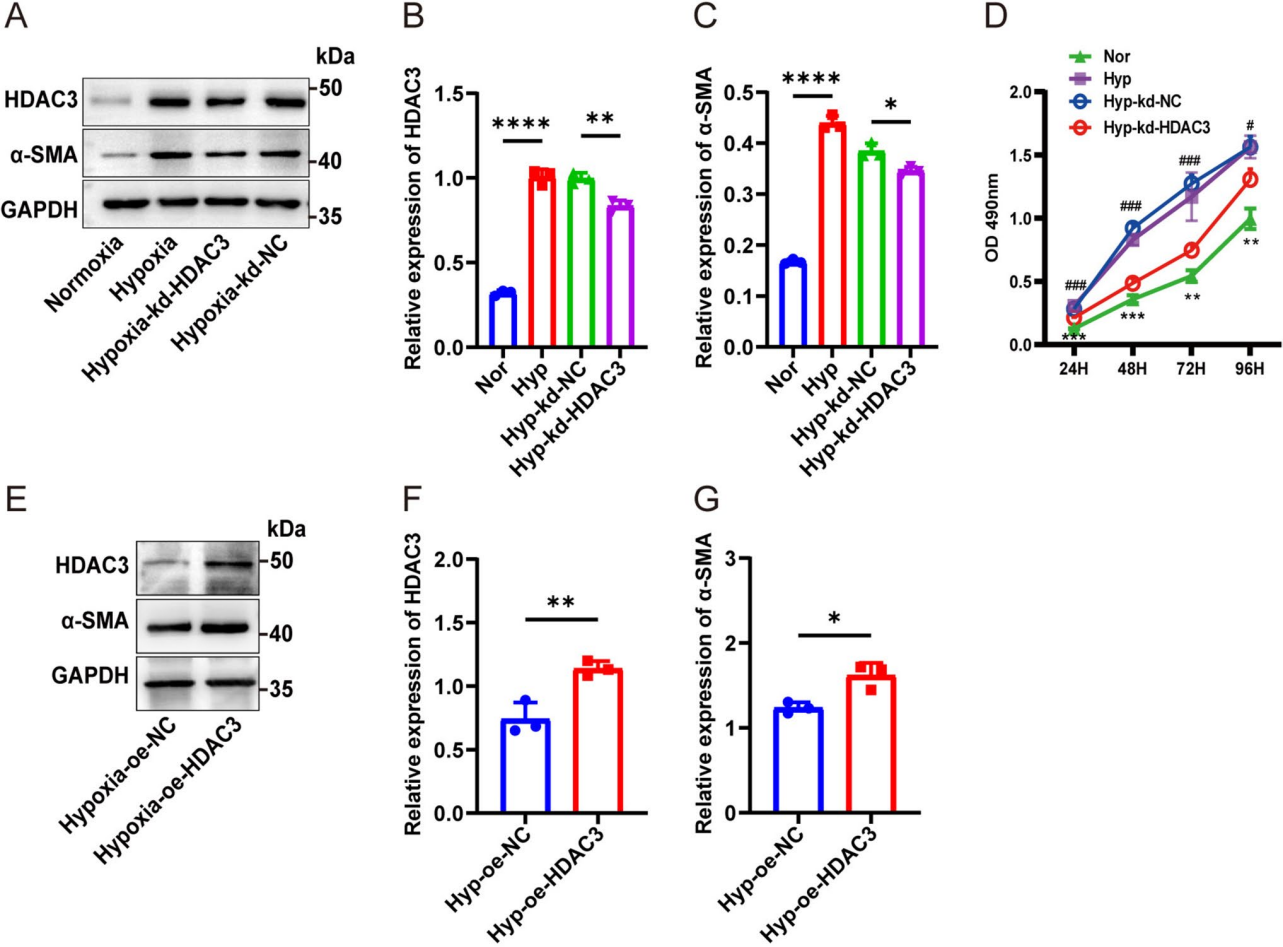
HDAC3 promoted hypoxia-induced hPASMCs’ proliferation. **A** hPASMCs were transfected with siRNA before hypoxia treatment, and western blotting was used to detect HDAC3 and α-SMA in cell lysates. **B**–**C** Protein levels of HDAC3 and α-SMA. **D** MTT assay of four groups. Data are expressed as the mean ± SD values. *P < 0.05, **P < 0.01, ***P < 0.001. *: Hyp group versus Nor group. ^#^*P* < 0.05, ^##^*P* < 0.01, ^###^*P* < 0.001 ^#^: Hyp-kd-NC group versus Hyp-kd-HDAC3 group. **E** hPASMCs were transfected with plasmid before hypoxia treatment, and western blotting was used to detect HDAC3 and α-SMA in cell lysates. **F**–**G** Protein levels of HDAC3 and α-SMA. The data are shown as the mean ± SD of at least three biologically independent replicates. Multiple-group comparisons were analyzed by one-way ANOVA followed by LSD post hoc test. *P < 0.05, **P < 0.01, ***P < 0.001. Nor normoxia, Hyp hypoxia, kd knockdown, NC negative control, oe overexpression

To further investigate the role of HDAC3 in hypoxia-induced hPASMC proliferation, HDAC3 knockdown and overexpression models were established. Transfection efficiency was confirmed by qRT-PCR (Supplementary Figs.  1 A and 1B). HDAC3 knockdown significantly reduced α-SMA protein levels under hypoxic conditions (Fig. 2A, C). Moreover, the MTT assay showed a time-dependent increase in HDAC3-knockdown cells under hypoxia relative to the hypoxia-only group (Fig. 2D).

To assess the influence of HDAC3 overexpression, hPASMCs were transfected with an HDAC3 overexpression plasmid and cultured under hypoxia. Moreover, α-SMA protein levels were elevated in HDAC3–overexpressing hPASMCs under hypoxia (Fig. 2E–G). These results collectively indicate that HDAC3 promotes hypoxia-induced hPASMC proliferation.

### Comparison of transcriptome profiles between HDAC3-KD and control groups under hypoxia

**Fig. 3:**
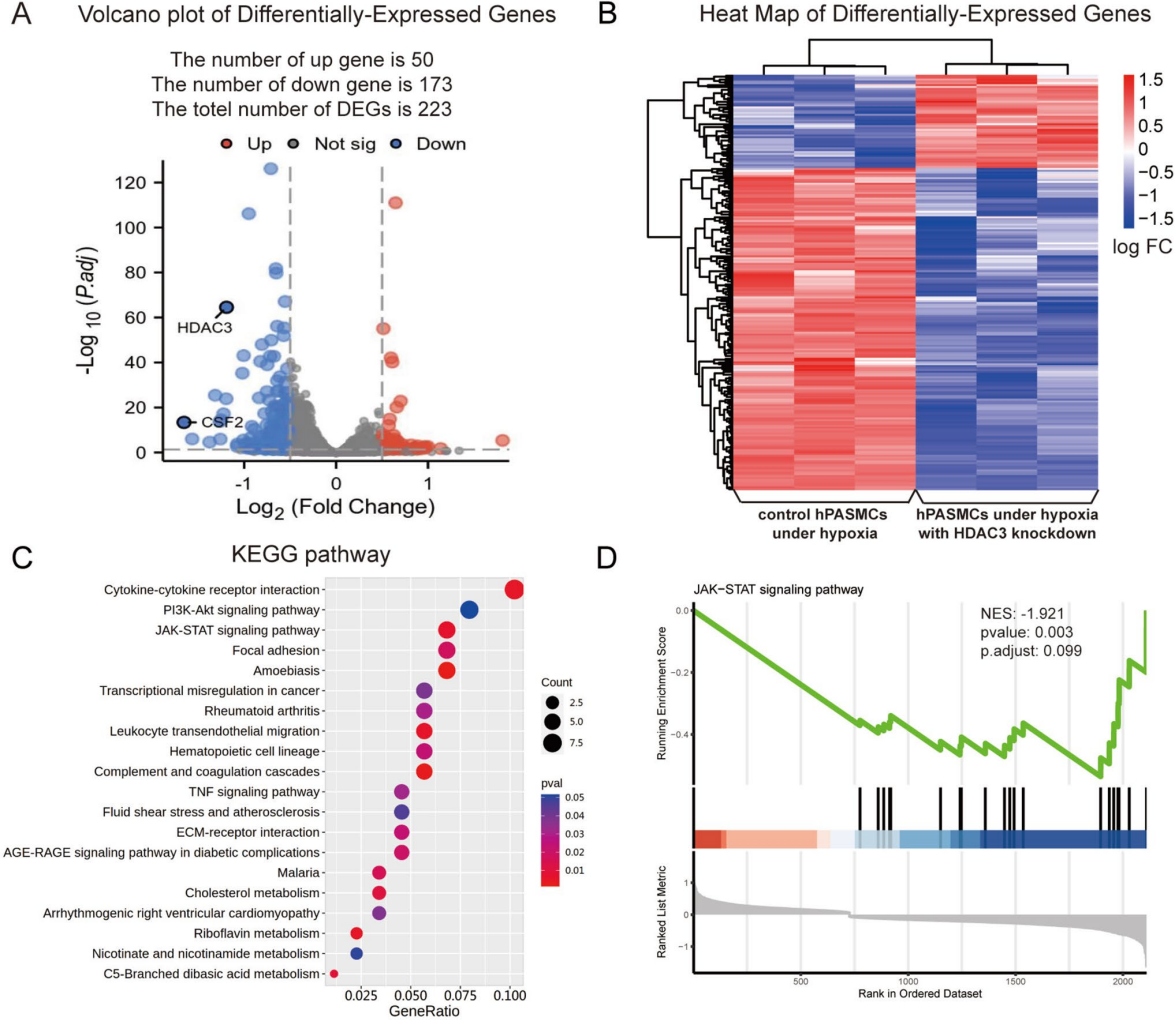
Transcriptome sequencing between HDAC3-KD group and KD-NC group under hypoxia. **A** Volcano plot of DEGs with |log2FC|> 1.5 and adjusted *p* value < 0.05. Upregulated and downregulated genes were colored red and blue, respectively. The gray spots indicate not significantly changed genes. **B** Heat map displaying the top 223 DEGs of RNA-seq. **C** 20 KEGG pathways were significantly enriched. The size and color of dots represent the count of genes and adjusted p value in the selected pathway. **D** JAK-STAT signaling pathway in GSEA term correlated with CSF2. hPASMCs: human pulmonary arterial smooth muscle cells

To elucidate the impact of HDAC3 knockdown on hypoxia-induced gene expression changes, transcriptome sequencing was performed on total RNA extracted from HDAC3-KD and NC-KD hPASMCs under hypoxia. A total of 223 DEGs (adjusted *P* < 0.05 and |log₂FC|> 1.5) were identified, including 50 upregulated and 173 downregulated genes in the HDAC3-KD group (HDAC3-KD versus NC-KD; Figs. 3A, B).

KEGG pathway enrichment analysis revealed that DEGs were significantly associated with cytokine–cytokine receptor interaction, PI3K-AKT signaling, and the JAK-STAT signaling pathway (Fig. 3C). GSEA further indicated the downregulation of the JAK-STAT pathway in the HDAC3-KD group (Fig. 3D and Table S1). Among genes enriched within the JAK-STAT pathway, *CSF2, CSF3, FHL1, PRLR,* and *LIF* were significantly downregulated in HDAC3-KD cells (Supplementary Figs. 3), suggesting a major role of this pathway in HDAC3's regulated hPASMC responses under hypoxia.

**Fig. 4:**
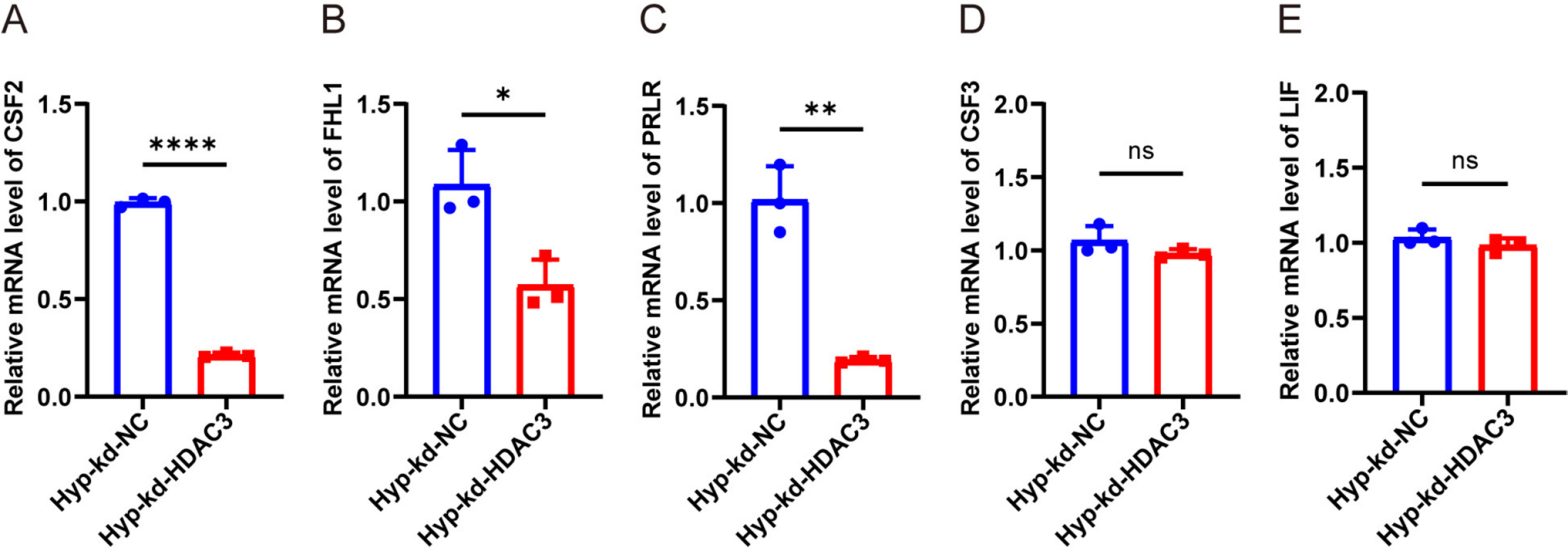
JAK/STAT pathway enrichment gene verification. The mRNA levels of CSF2 (**A**), FHL1 (**B**), PRLR (**C**), CSF3 (**D**), and LIF (**E**) between knockdown negative control and knockdown HDAC3 under hypoxia in hPASMCs. The data are shown as the mean ± SD of at least three biologically independent replicates. Comparisons were analyzed by Student’s t-test. ns: no significance. *P < 0.05, **P < 0.01, ***P < 0.001, ****P < 0.0001. Hyp hypoxia, kd knockdown, NC negative control

To verify the RNA-seq findings, qRT-PCR was conducted. The mRNA levels of *CSF2, FHL1,* and *PRLR* were markedly reduced in the HDAC3-KD group relative to the negative control under hypoxia (Fig. 4A–C). No significant differences were observed in *CSF3* or *LIF* expression (Fig. 4D, E), partially confirming the transcriptomic results.

### HDAC3 upregulated the CSF2 level and activated the JAK2/STAT5 pathway in hPASMCs under hypoxia

**Fig. 5:**
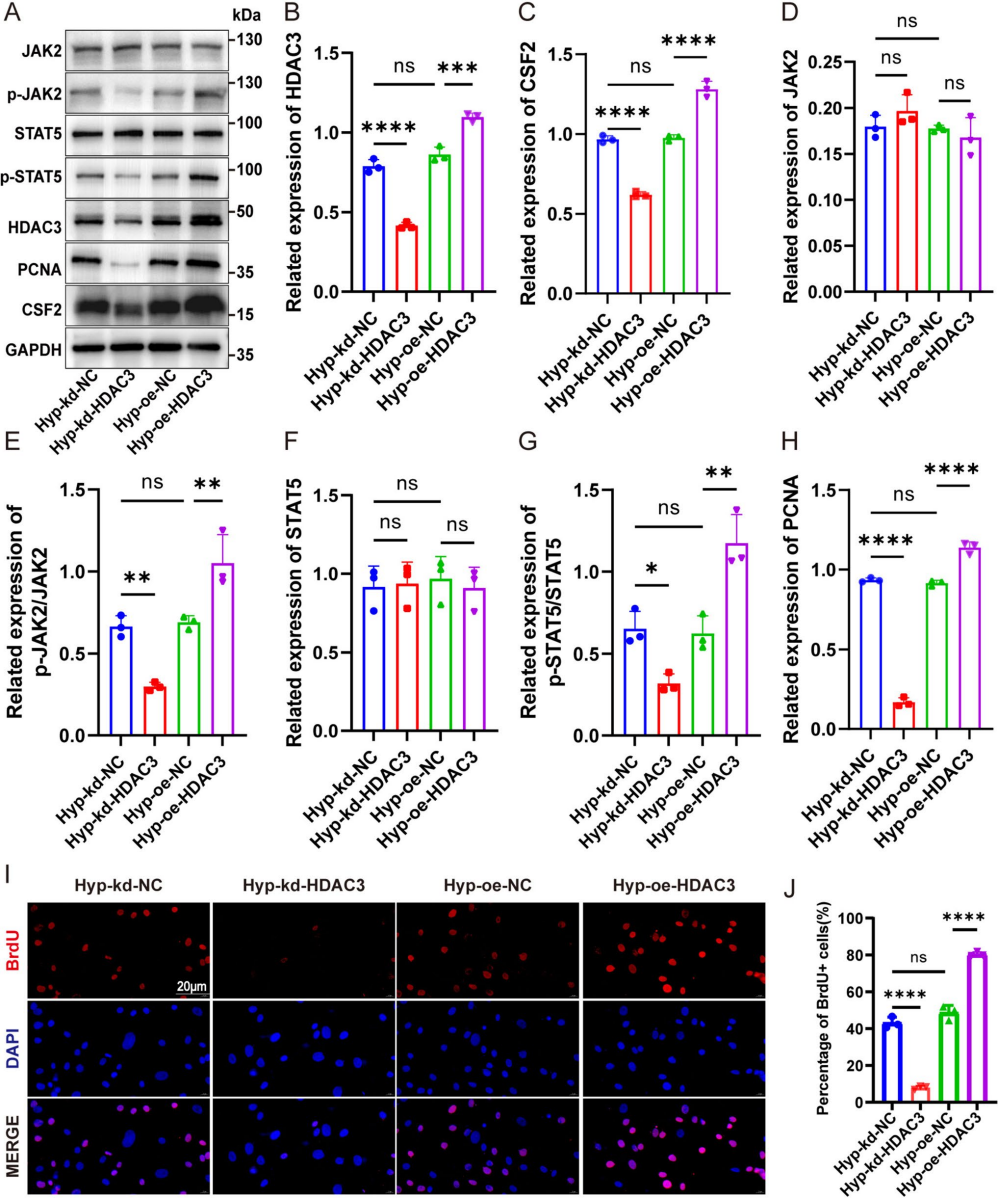
HDAC3 upregulated the CSF2 level and activated the JAK2/STAT5 pathway in PASMCs under hypoxia. **A** hPASMCs were transfected with si-HDAC3 or plasmid before hypoxia treatment, and western blotting was used to detect HDAC3, CSF2, PCNA, JAK2, P-JAK2, STAT5, and P-STAT5 in cell lysates. Protein levels of HDAC3 (**B**), CSF2 (**C**), PCNA (**H**), and JAK2/STAT5 pathway (**D**–**G**). **I**–**J** Immunofluorescent detection of BrdU-labeled cells (red) after knockdown or overexpression of HDAC3 under hypoxia condition in hPASMCs. The data are shown as the mean ± SD of at least three biologically independent replicates. Multiple-group comparisons were analyzed by one-way ANOVA followed by LSD post hoc test. s: no significance. *P < 0.05, **P < 0.01, ***P < 0.001, ****P < 0.0001. Hyp hypoxia, kd knockdown, NC negative control, oe overexpression

To further verify the transcriptomic findings, protein levels of CSF2, p-JAK2, and p-STAT5 were examined by western blotting. Knockdown of HDAC3 reduced the levels of CSF2, p-JAK2, and p-STAT5, whereas HDAC3 overexpression upregulated these proteins (Fig. 5A–G). Simultaneously, HDAC3 knockdown reduced the expression of PCNA and the ratio of BrdU-positive cells, indicating attenuated proliferation. On the other hand, HDAC3 overexpression increased both PCNA levels and BrdU-positive cell ratios under hypoxic conditions (Fig. 5H–J).

### HDAC3 activated the JAK2/STAT5 pathway via CSF2 to increase hPASMC proliferation under hypoxia

**Fig. 6:**
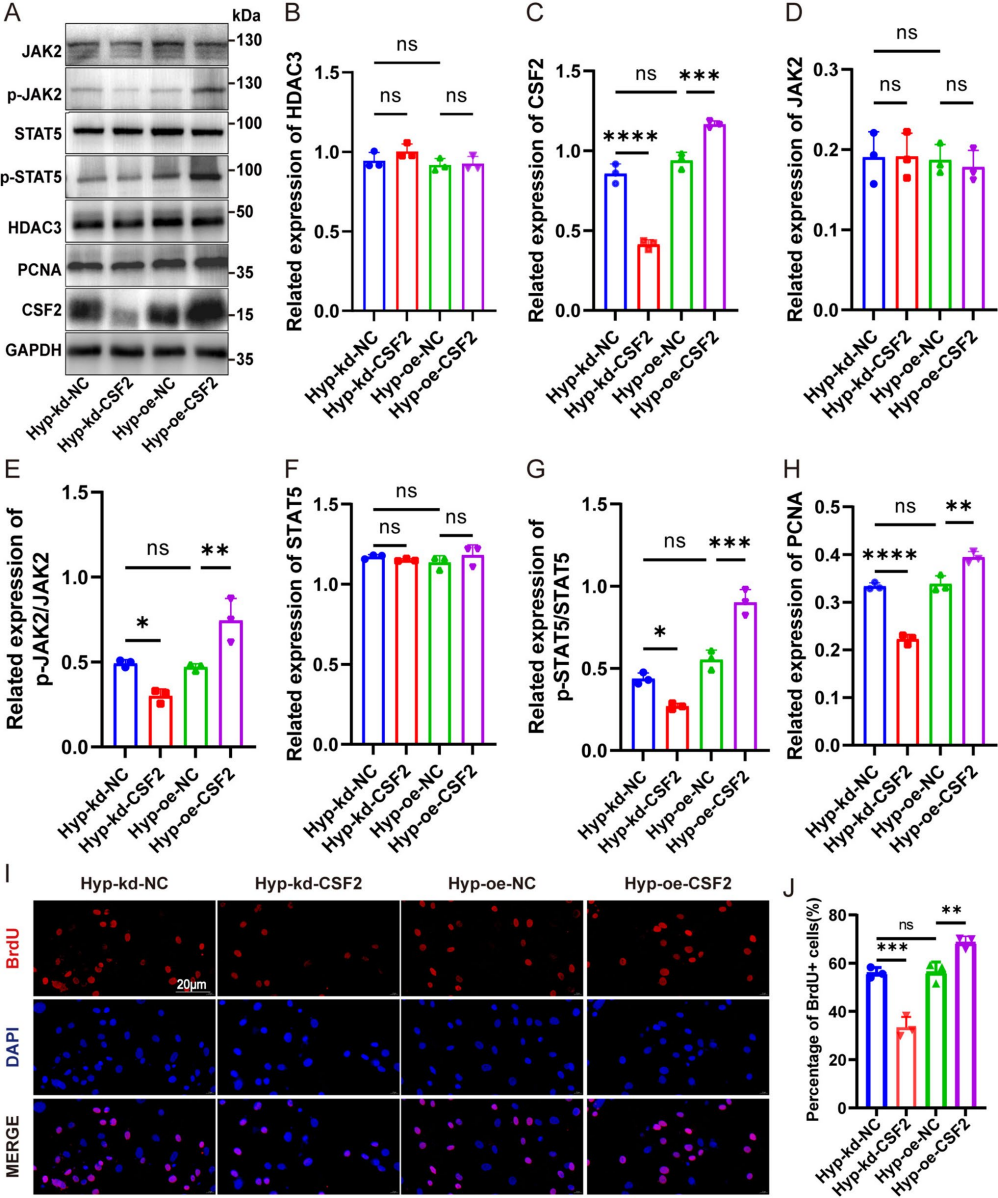
HDAC3 led to CSF2 dependent JAK2/STAT5 pathway activation causing enhancing proliferation in hPASMCs under hypoxia. **A** hPASMCs were transfected with si-CSF2 or plasmid before hypoxia treatment, and western blotting was used to detect HDAC3, CSF2, PCNA, JAK2, p-JAK2, STAT5, and *p*-STAT5 in cell lysates. Protein levels of HDAC3 (**B**), CSF2 (**C**), PCNA (**H**), and JAK2/STAT5 pathway (**D**–**G**). **I**–**J** Immunofluorescent detection of BrdU-labeled cells (red) after knockdown or overexpression of CSF2 under hypoxia condition in hPASMCs. The data are shown as the mean ± SD of at least three biologically independent replicates. Multiple-group comparisons were analyzed by one-way ANOVA followed by LSD post hoc test. s: no significance.*P < 0.05, **P < 0.01, ***P < 0.001, ****P < 0.0001. Hyp hypoxia, kd knockdown, NC negative control, oe overexpression

To further investigate the upstream and downstream relationship between HDAC3 and CSF2, hPASMCs with CSF2 knockdown and overexpression were established, with transfection efficiency confirmed by qRT-PCR (Supplementary Figs.  1 C, D). CSF2 knockdown reduced the hypoxia-induced elevation of p-JAK2 and p-STAT5 (Fig. 6C–G) without affecting HDAC3 protein levels (Fig. 6A, B). On the other hand, CSF2 overexpression elevated p-JAK2 and p-STAT5 levels. Moreover, CSF2 knockdown significantly inhibited HDAC3-induced proliferation of hPASMCs, while CSF2 overexpression restored it under hypoxia, as indicated by PCNA protein levels and BrdU-positive cell ratios (Fig. 6H–J). These findings indicate that CSF2 acts downstream of HDAC3 and upstream of the JAK2/STAT5 pathway, contributing to HDAC3-mediated proliferation in hPASMCs under hypoxic conditions.

To confirm whether CSF2 acts downstream of HDAC3, we established a "HDAC3 overexpression + CSF2 knockdown" co-intervention model in hPASMCs under hypoxia, seen in Supplementary Fig. 2. Compared with the Hyp-oe-HDAC3 group, the Hyp-oe-HDAC3-kd-CSF2 group significantly reduced the elevated p-JAK2/p-STAT5 and PCNA levels induced by HDAC3 overexpression (Supplementary Fig.  2 A and D–H), while the HDAC3 protein level remained unchanged across the two groups (Supplementary Figs.  2 A, B), confirming CSF2 does not affect HDAC3 expression. This result demonstrates that HDAC3-mediated activation of JAK2/STAT5 and promotion of proliferation are dependent on CSF2, supporting CSF2 as a downstream mediator of HDAC3.

### HDAC3 promoted hPASMCs proliferation via the CSF2/JAK2/STAT5 pathway under hypoxia

**Fig. 7:**
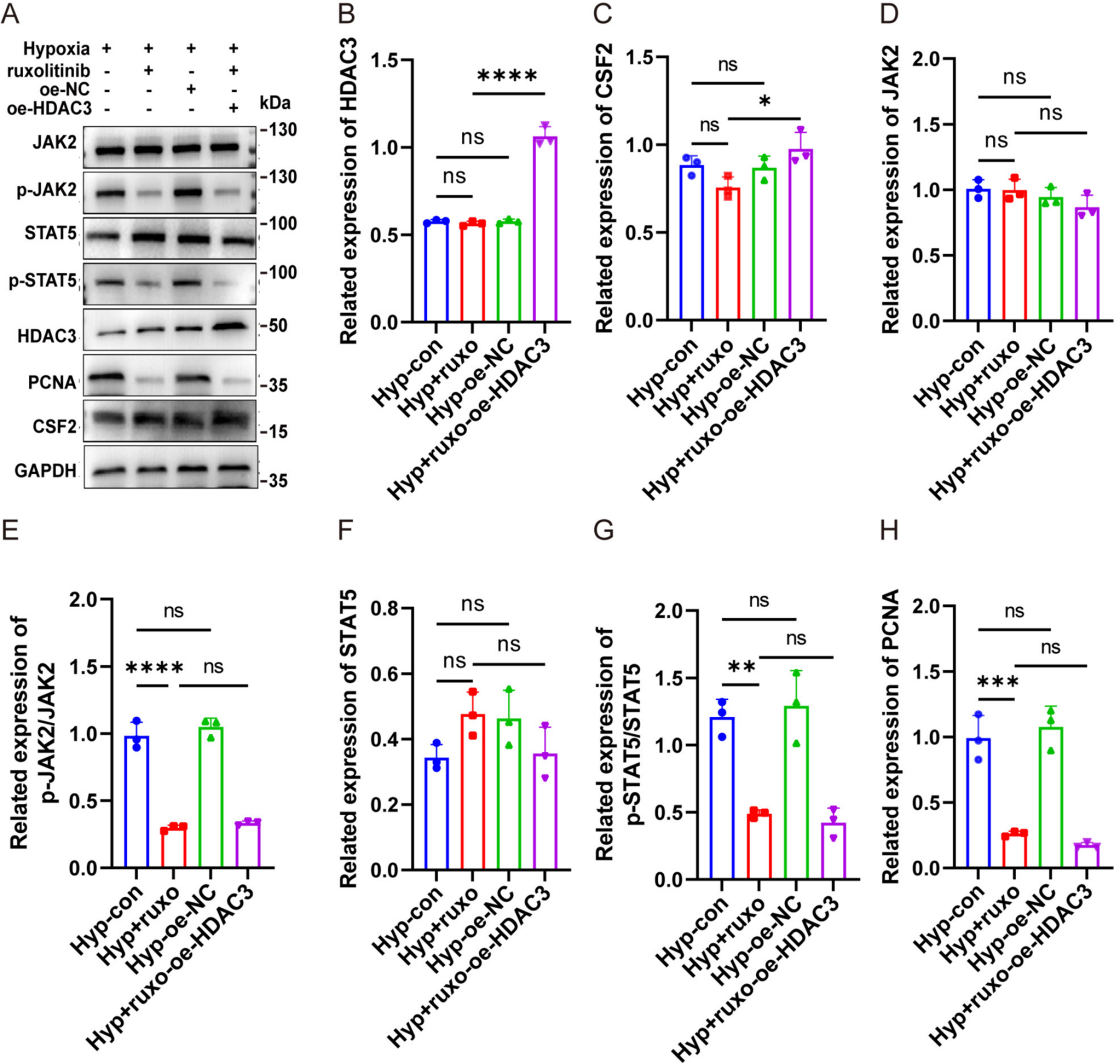
HDAC3 promoted PASMC proliferation via the CSF2/JAK2/STAT5 pathway under hypoxia. **A** hPASMCs were transfected with plasmid and treated with ruxolitinib for 24 h before hypoxia treatment; western blotting was used to detect HDAC3, CSF2, PCNA, JAK2, p-JAK2, STAT5, and p-STAT5 in cell lysates. Protein levels of HDAC3 (**B**), CSF2 (**C**), PCNA (**H**), and JAK2/STAT5 pathway (**D**–**G**). The data are shown as the mean ± SD of at least three biologically independent replicates. Multiple-group comparisons were analyzed by one-way ANOVA followed by LSD post hoc test. s: no significance. *P < 0.05, **P < 0.01, ***P < 0.001. Hyp hypoxia, kd knockdown, NC negative control, oe overexpression, ruxo ruxolitinib

To confirm the involvement of the CSF2/JAK2/STAT5 axis, JAK2 kinase activity was pharmacologically inhibited using ruxolitinib, a selective JAK1/2 inhibitor under clinical investigation for various malignancies [28]. Ruxolitinib treatment inhibited hypoxia-induced PCNA expression (Fig. 7H) while decreasing the levels of p-JAK2 and p-STAT5 (Figs. 7D–G). Ruxolitinib did not alter the protein levels of HDAC3 or CSF2 (Figs. 7A–C), indicating that its inhibitory effects occurred downstream of CSF2. Overexpression of HDAC3 failed to reverse the reduction in p-JAK2 and p-STAT5 levels induced by ruxolitinib treatment. Collectively, these data demonstrate that HDAC3 promotes hPASMC proliferation under hypoxia through the CSF2-mediated activation of the JAK2/STAT5 signaling pathway, implicating this axis in the vascular remodeling process observed in PAH.

## Discussion

**Fig. 8:**
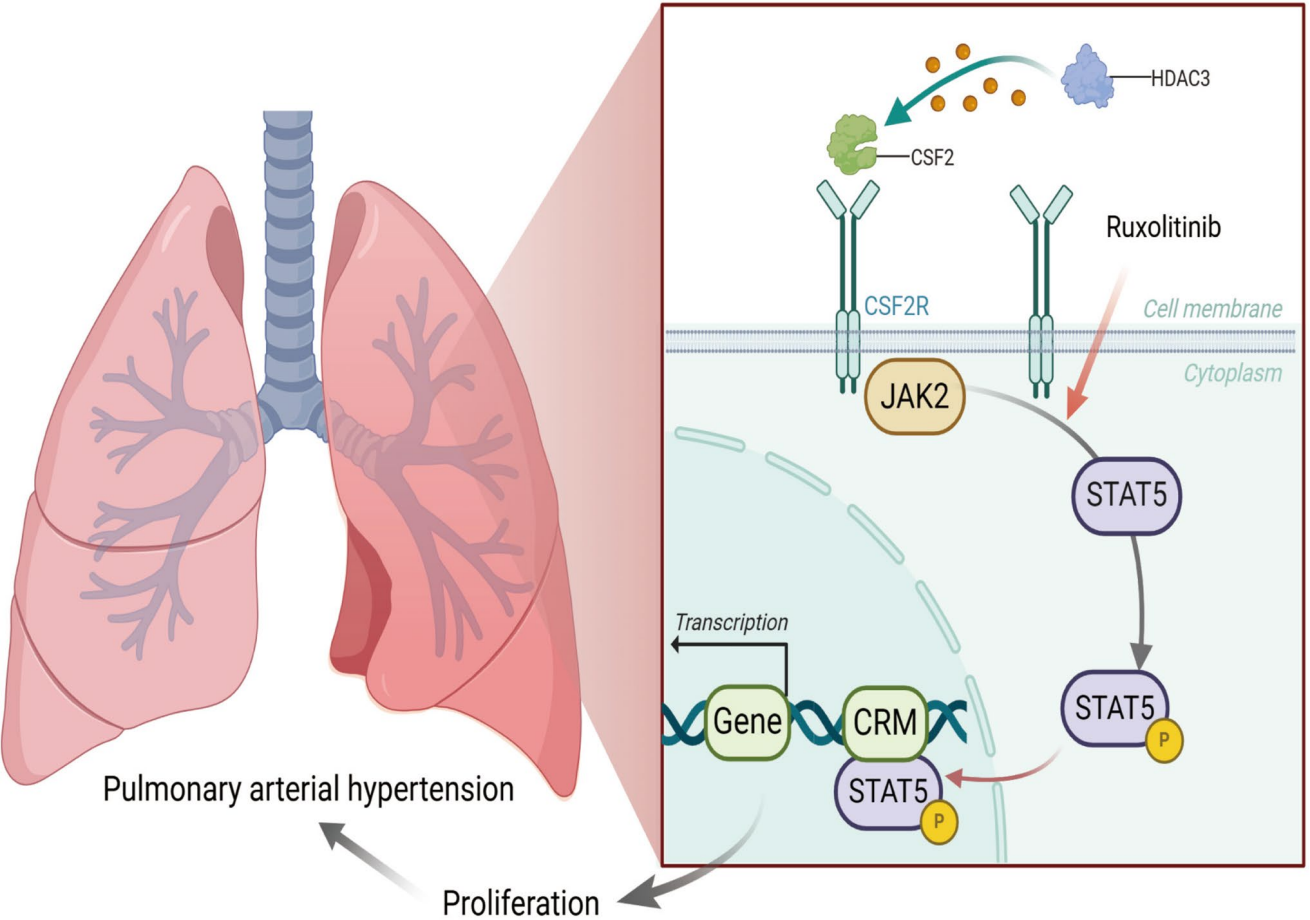
Proposed mechanism by which HDAC3 regulates human pulmonary arterial smooth muscle cells' proliferation

In this study, hypoxia-induced hPASMC proliferation models were used to investigate the association between HDAC3 and the activation of the CSF2/JAK2/STAT5 signaling pathway. The results indicated that hypoxia significantly increased hPASMC proliferation, accompanied by increased HDAC3 mRNA and protein expression. HDAC3 knockdown effectively inhibited hypoxia-induced hPASMC proliferation. Moreover, CSF2, p-JAK2, and p-STAT5 levels were significantly upregulated in response to hypoxia, and this upregulation was reversed by HDAC3 siRNA, CSF2 siRNA, or the JAK2 inhibitor ruxolitinib (Fig. 8). To our knowledge, this is the first study demonstrating that HDAC3 promotes hPASMC proliferation under hypoxia by activating the CSF2/JAK2/STAT5 pathway.

It is well known that various regulatory genes and cytokines cause the progression of PAH, which involves complex pathological mechanisms. Hypoxia, a key environmental factor, has been shown to exert widespread effects on cellular physiology, including the modulation of proliferation and inflammation-related processes [29–31]. Consistent with previous findings, this study confirmed that hypoxia induces excessive proliferation of hPASMCs, a hallmark of pulmonary vascular remodeling in PAH.

HDAC3, a catalytic component of the HDAC3/NCoR/SMRT repressor complex, functions through both enzymatic and non-enzymatic mechanisms. Recruitment of HDAC3 into this complex increases its deacetylase activity, allowing it to repress transcription in association with various transcriptional regulators [9, 10, 32]. Beyond its classical roles, HDAC3 also exhibits non-enzymatic functions relevant to transcriptional regulation [9, 10, 33]. However, its role in hPASMC biology remains inadequately defined. Only two previous studies have addressed the direct involvement of HDAC3 in hPASMCs, and their conclusions differ. Eva NG et al. (2016) reported that HDAC3 regulates SOD3 expression in hPASMCs from patients with IPAH, where HDAC3 knockdown, but not HDAC1 or HDAC2, inhibited cell proliferation and upregulated SOD3 expression, supporting its therapeutic potential [9]. In contrast, Prakash C et al. found increased expression of HDAC1 and HDAC8, but not HDAC3, in pulmonary arteries from IPAH patients [34]. In the present study, HDAC3 was significantly upregulated in hPASMCs under hypoxic conditions, and its inhibition mitigated hypoxia-induced proliferation, aligning with the findings by Eva NG et al. [9] and supporting the relevance of HDAC3 in pulmonary vascular remodeling in PAH. In our RNA-sequencing data, HDAC3 knockdown alters the expression of 223 genes, including key proliferation-related factors and JAK/STAT pathway components. Conversely, HDAC3 overexpression amplifies the transcription of these pro-proliferative targets. This epigenetic cascade converts a small change in HDAC3 levels into a large-scale shift in cell signaling, ultimately driving robust proliferation.

Hyperproliferation and aberrant migration of hPASMCs under hypoxia contribute to PAH progression. Inhibition of hPASMC proliferation has been shown to alleviate disease pathology. In this context, HDAC3 knockdown significantly reduced cell proliferation, whereas HDAC3 overexpression increased it under hypoxia. These results suggest that HDAC3 is directly involved in regulating the proliferative response of hPASMCs to hypoxia. HDAC3 has been found to modulate inflammation through various pathways [7, 10]. Cytokines may serve as intermediaries in the HDAC3-mediated regulation of hPASMC proliferation. Previous research has shown that HDAC3 regulates inflammatory responses through multiple signaling pathways, including those involving Th1/Th17 cytokines. Various cellular and animal studies have shown that HDAC inhibition reduces the IL-1β, IL-6, and TNF-α expression [32, 35]. In this study, HDAC3 was found to regulate downstream targets, including CSF2, FHL1, and PRLR, all of which have been linked to PAH and acute lung injury pathogenesis [20, 21, 36]. CSF2 knockdown reversed the HDAC3-induced proliferative phenotype in hPASMCs under hypoxia. These results suggest that CSF2 functions as a key mediator of HDAC3 signaling in this context. The present study appears to be the first to show that CSF2 is required for HDAC3-mediated regulation of hPASMC proliferation under hypoxic conditions.

CSF2 is primarily secreted by activated monocytes, macrophages, fibroblasts, smooth muscle cells, endothelial cells, and various cancer cell types. It regulates multiple signaling pathways, promotes cellular proliferation and differentiation, and plays a role in various diseases [21, 31, 37]. In this study, CSF2 downregulation and the consequent inactivation of the JAK2/STAT5 pathway were identified as key mechanisms underlying the inhibition of hPASMC proliferation following HDAC3 knockdown. Similar regulatory patterns have been reported in other pathological contexts, including cancer [37, 38], but have not previously been described in hypoxia-induced hPASMC proliferation.

Several inflammatory mediators signal through the JAK/STAT pathway in response to hypoxia [13, 23, 39]. Recent evidence indicates that this pathway is overactivated in the pulmonary arteries of patients with various forms of pulmonary hypertension [14, 16, 19, 23]. JAK2 is also known to mediate signaling transduction from the IL-3 receptor family, including IL-3R, IL-5R, and the CSF2 receptor; therefore, coordinating proliferation-related responses. In this study, pharmacological inhibition of JAK2 with ruxolitinib effectively reduced hypoxia-induced hPASMC proliferation, highlighting the critical role of JAK2 in this context. Furthermore, the knockdown of either HDAC3 or CSF2, as well as JAK2 inhibition, resulted in reduced phosphorylation of STAT5. STAT5 deficiency has been associated with endoplasmic reticulum (ER) stress, nuclear morphological abnormalities, and disrupted ER-Golgi function—features implicated in the pathogenesis of PAH through genomic and novel nongenomic mechanisms [40, 41].

Several limitations in this study need to be addressed. Although the current data show that HDAC3 inhibition ameliorates hPASMC proliferation by inactivating the CSF2/p-JAK2/p-STAT5 pathway, whether HDAC3 regulates CSF2 expression directly or indirectly under hypoxic conditions warrants further investigation. In addition, except for PCNA and Brdu, other indicators such as Ki67 need to be detected in our future work.

In conclusion, this study proposed and validated a novel mechanism contributing to pulmonary vascular remodeling, identifying HDAC3 as a key regulator of hPASMC proliferation through the CSF2/JAK2/STAT5 signaling axis under hypoxia. While HDAC3 has been previously implicated in cytokine regulation and inflammatory signaling, such as the regulation of NF-κB in atherosclerosis and pulmonary injury [32, 42], its role in pulmonary vascular cell proliferation has not been clearly defined. The study findings provide new insights into the interactions between HDAC3 and inflammatory cytokines and the role of HDAC3 inhibition in controlling the hPASMCs’ proliferation via CSF2/JAK2/STAT5 signaling during hypoxia conditions in vitro, offering a potential therapeutic target. Further exploration of HDAC3 inhibition as a strategy to suppress hypoxia-induced hPASMC proliferation and improve PAH prognosis remains an important direction for future research.

## Supplementary Information

Below is the link to the electronic supplementary material.

Supplementary file1 (DOCX 16 KB)

Supplementary file2 (TIF 9498 KB)

Supplementary file3 (TIF 12093 KB)

Supplementary file4 (TIF 10102 KB)

Supplementary file5 (TIF 1012759 KB)

## Data Availability

The data generated in the present study may be requested from the corresponding author.
