# Supplementary material for: Histone deacetylase 3 promotes hypoxia-induced human pulmonary arterial smooth muscle cell proliferation by modulating the CSF2-JAK2-STAT5 signaling pathway
Source: Hum Cell. 2026 Jan 16;39(2):35. doi: 10.1007/s13577-026-01348-6 (PMC12811372; doi:10.1007/s13577-026-01348-6)
Supplement: Supplementary file 1 — Supplementary file1 (DOCX 16 KB) [file 13577_2026_1348_MOESM1_ESM.docx]

Supplementary Table1 The top 20 pathways of KEGG enrichment in GSEA

| **ID** | **Description** | **setSize** | **NES** | **pvalue** | **qvalue** |
| --- | --- | --- | --- | --- | --- |
| hsa03010 | Ribosome | 31 | 3.14365207995397 | 8.32154185357966e-09 | 2.63661483992366e-06 |
| hsa05168 | Herpes simplex virus 1 infection | 57 | 2.4481406489184 | 1.04787479219167e-06 | 0.000166005427605101 |
| hsa04514 | Cell adhesion molecules | 22 | -2.164923458 | 0.0001632005213421 | 0.012927199190519 |
| hsa04060 | Cytokine-cytokine receptor interaction | 28 | -2.131973621 | 0.000129447399075319 | 0.012927199190519 |
| hsa04610 | Complement and coagulation cascades | 12 | -2.069017696 | 0.000685279679312866 | 0.0434250912575101 |
| hsa05322 | Systemic lupus erythematosus | 8 | 2.06923142855334 | 0.00196457723149086 | 0.0918396353239583 |
| hsa04979 | Cholesterol metabolism | 7 | -1.963186885 | 0.00253222366693488 | 0.0918396353239583 |
| hsa04976 | Bile secretion | 7 | -1.940244732 | 0.00336423993177 | 0.0918396353239583 |
| hsa04640 | Hematopoietic cell lineage | 14 | -1.922222135 | 0.00265716282368111 | 0.0918396353239583 |
| hsa04630 | JAK-STAT signaling pathway | 20 | -1.920807034 | 0.0034783117697446 | 0.0918396353239583 |
| hsa05323 | Rheumatoid arthritis | 22 | -1.892143516 | 0.00291205714524177 | 0.0918396353239583 |
| hsa04940 | Type I diabetes mellitus | 6 | -1.87298715 | 0.00331092019710862 | 0.0918396353239583 |
| hsa00970 | Aminoacyl-tRNA biosynthesis | 12 | 1.95425792942168 | 0.00420927784621831 | 0.102590496494875 |
| hsa04657 | IL-17 signaling pathway | 28 | -1.780292082 | 0.00554417612023332 | 0.125473459563175 |
| hsa04927 | Cortisol synthesis and secretion | 7 | -1.837393488 | 0.0102291774930642 | 0.202564633250811 |
| hsa04015 | Rap1 signaling pathway | 43 | -1.672681628 | 0.0096373711732243 | 0.202564633250811 |
| hsa00910 | Nitrogen metabolism | 3 | -1.606425645 | 0.0108820923074256 | 0.202817943314868 |
| hsa04080 | Neuroactive ligand-receptor interaction | 17 | -1.748937006 | 0.0133505692525733 | 0.225876248481491 |
| hsa04151 | PI3K-Akt signaling pathway | 74 | -1.56833335 | 0.0135450707145878 | 0.225876248481491 |
| hsa03040 | Spliceosome | 16 | 1.77452051554152 | 0.0152379321256734 | 0.241400924727773 |
