# Supplementary figures and images for: Histone deacetylase 3 promotes hypoxia-induced human pulmonary arterial smooth muscle cell proliferation by modulating the CSF2-JAK2-STAT5 signaling pathway
Source: Hum Cell. 2026 Jan 16;39(2):35. doi: 10.1007/s13577-026-01348-6 (PMC12811372; doi:10.1007/s13577-026-01348-6)

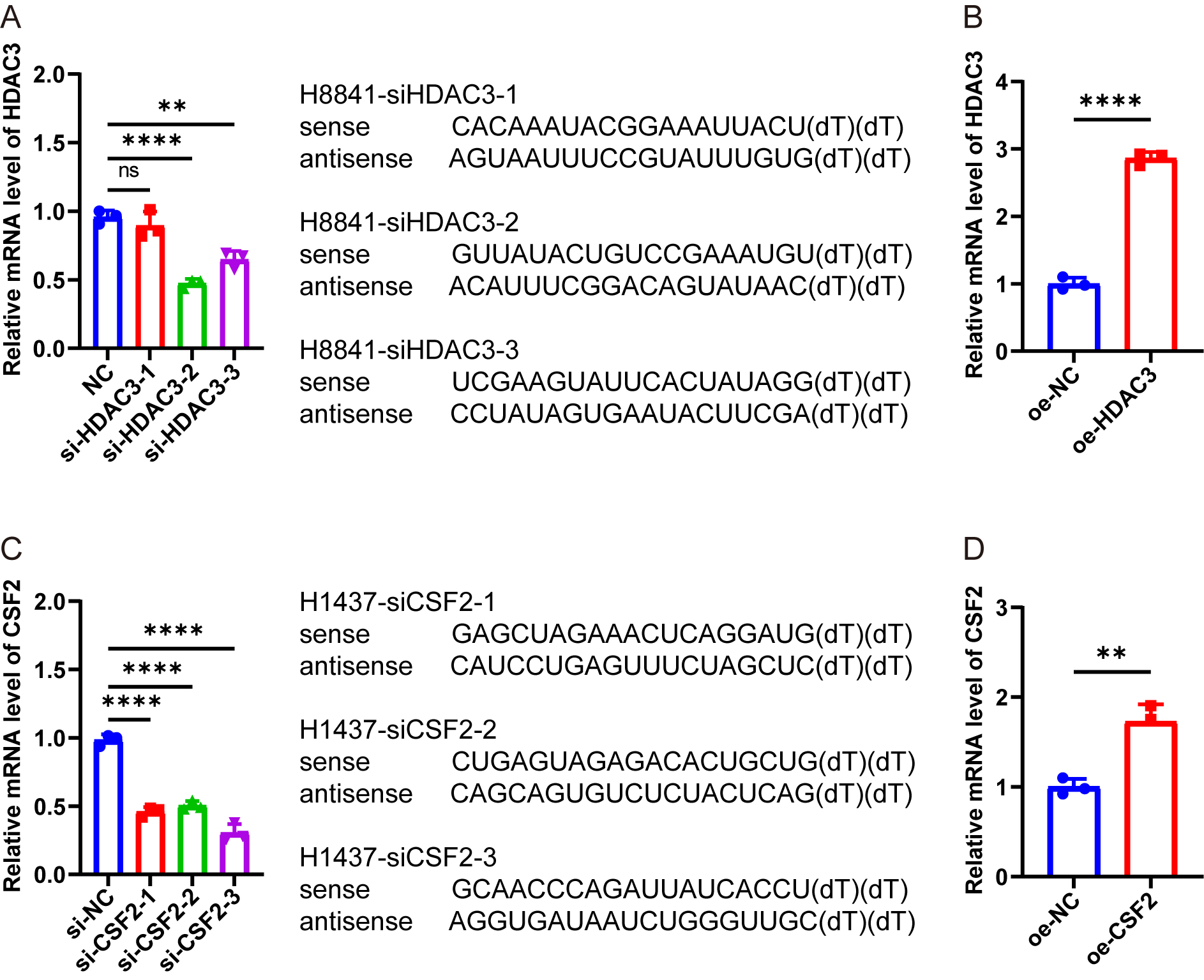

Supplement: Supplementary file 2 — Supplementary file2 (TIF 9498 KB) [file 13577_2026_1348_MOESM2_ESM.tif]

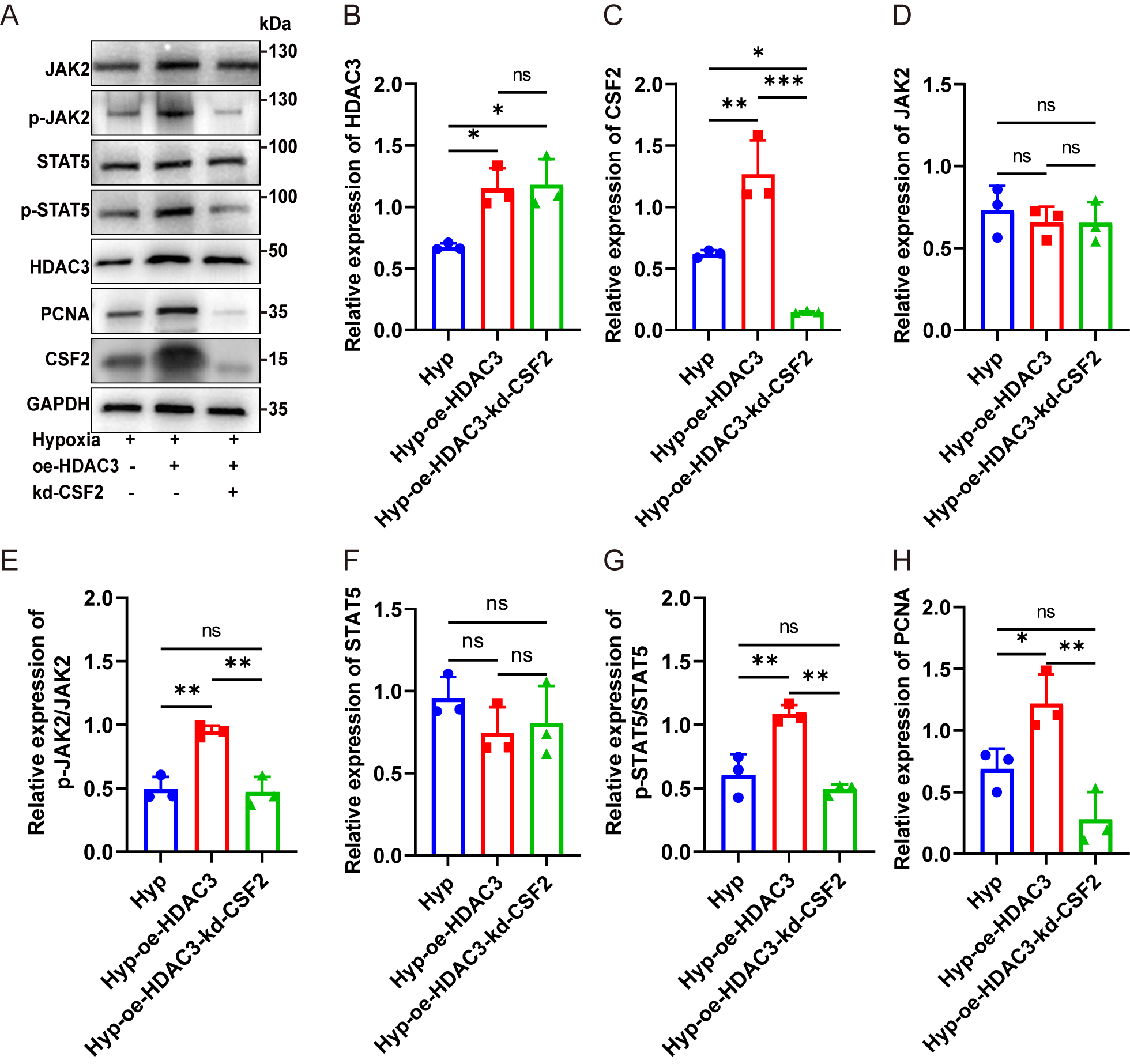

Supplement: Supplementary file 3 — Supplementary file3 (TIF 12093 KB) [file 13577_2026_1348_MOESM3_ESM.tif]

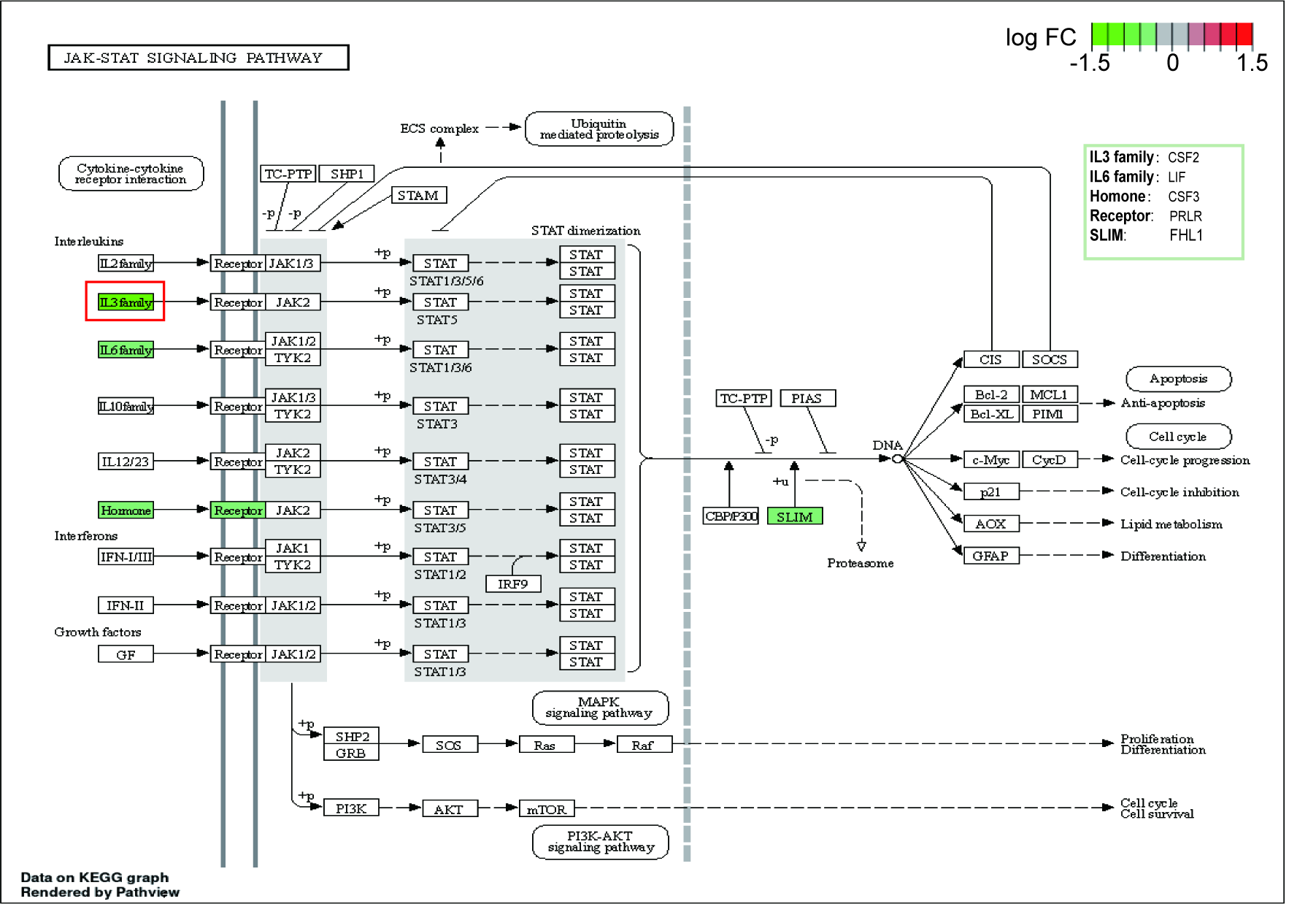

Supplement: Supplementary file 4 — Supplementary file4 (TIF 10102 KB) [file 13577_2026_1348_MOESM4_ESM.tif]
